# Intra-articular Plica Causing Ankle Impingement Syndrome: A Case Report and Updated Review of the Literature

**DOI:** 10.7759/cureus.100205

**Published:** 2025-12-27

**Authors:** Sarah Oyadomari, Henry Avetisian, Justin C Haghverdian, Andrew R Hsu, Naudereh Noori

**Affiliations:** 1 Orthopedic Surgery, University of California Irvine Medical Center, Orange, USA; 2 Orthopedic Surgery, Jacobs School of Medicine and Biomedical Sciences, University of Buffalo, Buffalo, USA

**Keywords:** ankle arthroscopy, ankle impingement, ankle plica syndrome, chronic ankle pain, intra-articular plica

## Abstract

Ankle plica syndrome is a rare and underrecognized cause of chronic ankle pain, often overlooked due to nonspecific clinical and imaging findings. We present the case of a 17-year-old female athlete with ankle pain and mechanical symptoms refractory to conservative treatment who was diagnosed with intra-articular ankle plica syndrome and successfully treated with extensive arthroscopic debridement. We also share a review of the current literature on symptomatic ankle plica syndrome. This case contributes to the limited existing literature and underscores the importance of considering this diagnosis in patients with chronic, activity-limiting ankle pain unresponsive to conservative management.

## Introduction

Plicae are synovial folds that arise from the incomplete resorption of embryologic septa within joint cavities during development. Although often asymptomatic and typically identified incidentally during arthroscopy, plicae can become clinically significant under certain conditions. Repetitive microtrauma, mechanical irritation, or overuse may lead to inflammation, fibrosis, and subsequent impingement, resulting in plica syndrome [1]. While this phenomenon is well-documented in the knee, plica syndrome in the ankle is not well described. Due to its low prevalence and nonspecific imaging features, ankle plicae are often overlooked as a potential cause of chronic ankle pain, mechanical symptoms, or anterior impingement. 

In this report, we present the case of a 17-year-old female athlete with persistent anterior ankle pain and locking, ultimately found to have an intra-articular ankle plica. The patient was successfully treated with arthroscopic debridement after failing conservative measures. We also provide a review of the current literature on intra-articular ankle plicae, highlighting their clinical presentation, diagnostic challenges, and treatment outcomes. 

## Case presentation

A 17-year-old female athlete with no significant medical history presented with a two-month history of atraumatic dull pain localized to the left ankle. The pain worsened with weight-bearing and persisted despite a course of conservative management including rest, nonsteroidal anti-inflammatory drugs (NSAIDs), physical therapy, bracing, and taping. She was unable to return to full sports activity due to the symptoms.

**Figure 1:**
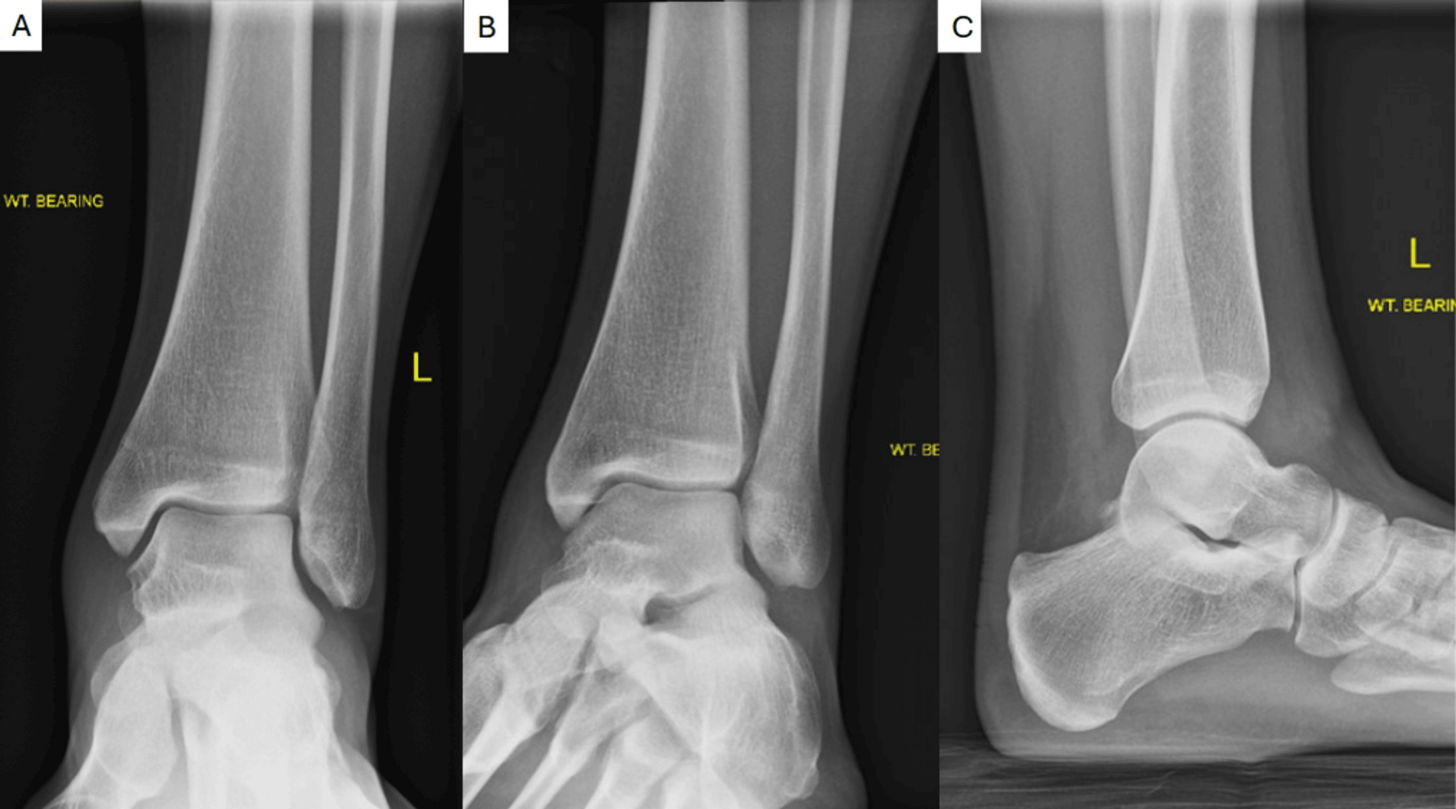
Anteroposterior (A), oblique (B), and lateral (C) weightbearing radiographs of the left ankle.

**Figure 2:**
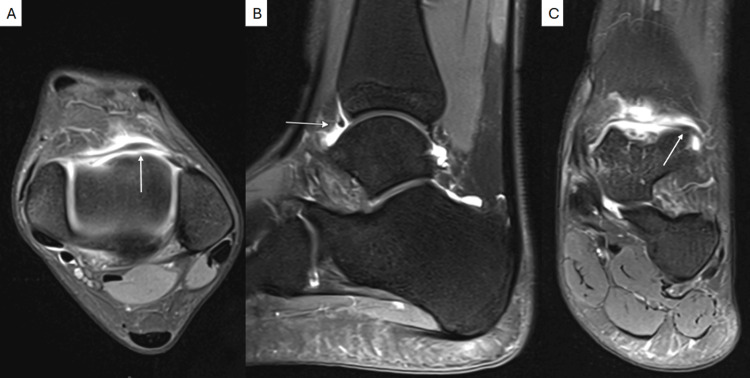
Axial (A), sagittal (B), and coronal (C) proton density fat-saturated MRI images. The intra-articular plica is denoted by the white arrows.

On examination, she had neutral ankle and hindfoot alignment, full ankle and hindfoot range of motion, and normal motor strength throughout the extremity. There was notable swelling about the ankle with pain at the anterior ankle with palpation and with terminal dorsiflexion. There was no appreciable laxity. Weight-bearing ankle radiographs were unremarkable (Figure 1). Magnetic resonance imaging (MRI) revealed a small, approximately 4 × 4 mm soft tissue focus within the anterior ankle joint, suggestive of thickened synovium or a plica (Figure 2). There was no evidence of osteochondral lesions or ligamentous injury on MRI imaging. Given persistent symptoms refractory to a course of conservative treatment, ankle arthroscopy with extensive debridement was performed. 

**Figure 3:**
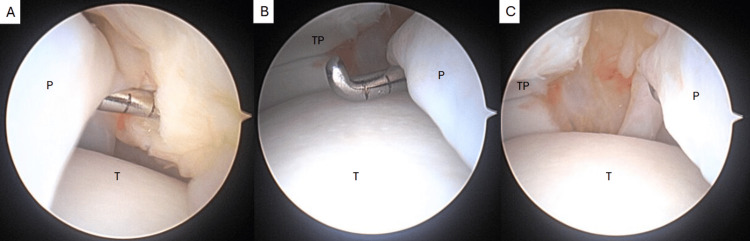
Arthroscopic images of the ankle plica viewed from the anteromedial portal showing anterior to the plica (A) and posterior to the plica (B). Probe hooked under the plica showing it draped across the talus from medial to lateral (C). P: plica, T: talus, TP: tibial plafond.

**Figure 4:**
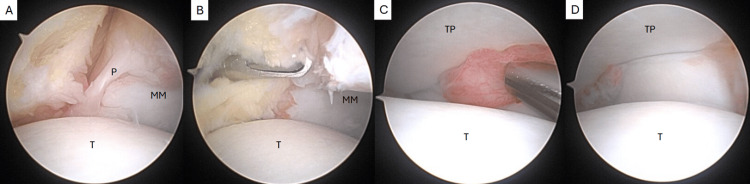
Anteromedial ankle with additional plica band noted extending from medial tibial plafond to the medial gutter (A) debrided (B). Lateral ankle and syndesmosis with extensive synovitis (C) debrided (D). P: plica, T: talus, MM: medial malleolus, TP: tibial plafond

**Figure 5:**
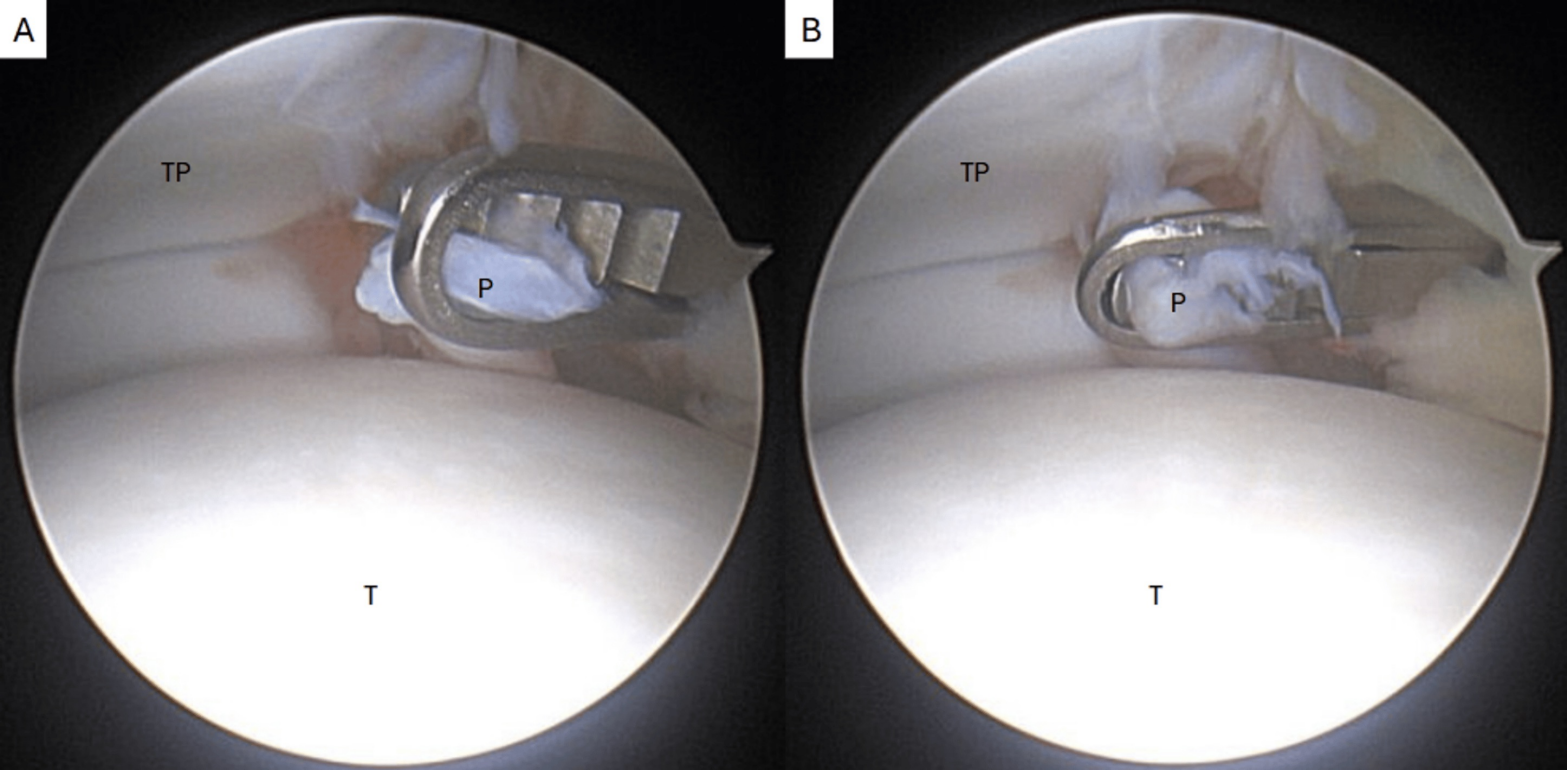
Debridement of the plica using an arthroscopic biter. P: plica, T: talus, TP: tibial plafond.

**Figure 6:**
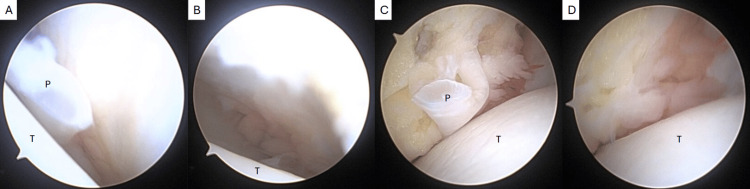
Lateral gutter with residual plica (A) debrided (B). Medial gutter with residual plica (C) debrided (D). P: plica, T: talus.

Arthroscopy revealed a prominent thick band of soft tissue draping across the anterior ankle joint that was continuous from the medial to lateral gutters (Figure 3, Video 1). The band measured approximately 4 mm in thickness, which correlated with the preoperative MRI. There was extensive synovitis at the syndesmosis as well as anterolateral and anteromedial aspects of the ankle with no chondromalacia (Figure 4). Along the anteromedial aspect of the ankle, there was noted to be an additional band extending from the medial tibial plafond to the medial gutter, forming a Y-shaped structure with the primary plica (Figure 4A). The entire plica was removed using a combination of an arthroscopic biter and 3.5 mm shaver with care to ensure the entirety of the band was resected from the gutters (Figures 5, 6). The synovitis was debrided using the shaver, and final visualization of the medial and lateral gutters confirmed no residual free fragments or entrapped soft tissue (Figures 4, 6). 

**Video 1:** Arthroscopy showing medial to lateral plica in ankle joint viewed from the anteromedial portal.

The patient was allowed to be weight-bearing as tolerated in a soft dressing and a walking boot immediately after surgery. She was seen in the clinic for suture removal two weeks postoperatively. At six weeks postoperatively, she reported significant improvement in her symptoms and had returned to all activities, including jogging and soccer. At six months postoperatively, she continued to report no recurrence of pain, mechanical symptoms, or feelings of instability after having returned to playing soccer.

## Discussion

While synovial plica syndrome is well described within the knee, it is less frequently identified as a source of pain in the ankle. Due to nonspecific clinical exam findings and inconclusive MRI findings, ankle plica syndrome may often be overlooked or underdiagnosed. The earliest reports of ankle plica in the literature were in cohorts investigating arthroscopy techniques for diagnostic purposes and treatment of synovitis [2-4]. The plicae identified in these patients were laterally based and did not seem to span the ankle joint. There was minimal detail on the presentation of these patients and specific findings. Review of the literature identified three later published case reports reporting a total of four cases of symptomatic ankle plica due to intra-articular synovial folds (Table 1). 

**Table 1:** Summary of published case reports on ankle plicae. ATFL: anterior talofibular ligament, PT: physical therapy, NSAID: non-steroidal anti-inflammatory drug, XR: radiograph, MRI: magnetic resonance imaging.

Case Report	Pertinent History	Presentation	Imaging	Arthroscopy Findings	Treatment	Postoperative Course
Highcock et al., 2012 [5]	16-year-old male soccer player with no history of trauma	Eighteen months of pain and snapping over anteromedial ankle. Audible click with dorsiflexion.	XR: Unremarkable; MRI: Unremarkable; MRI arthrogram: thickened synovium fold anteromedially deep to tibialis anterior tendon	Synovial plica in anteromedial ankle with adjacent chondromalacia on anterior talus	Arthroscopic debridement with a burr	Full weight-bearing in boot, return to full sport activities at 12 weeks
Somorjai et al., 2013 [6]	16-year-old male handball player with multiple minor inversion injuries	Three years of intermittent pain and locking in ankle. Tender over ATFL.	XR: Unremarkable; MRI: Flake fracture/loose body at the anteromedial talar dome	Soft tissue structure between tibial plafond (osteochondral fossa) and medial malleolus	Arthroscopic debridement with a shaver	Partial weightbearing, return to sport activities at 6 weeks
Rosenbaum et al., 2015 [7]	34-year-old female marathoner with repeated ankle sprains treated conservatively	One year of intermittent, sharp pain at anterolateral ankle, refractory to rest, PT, NSAIDs, steroid injection. Pain with forced dorsiflexion.	XR: Unremarkable; MRI: Synovitis in anterolateral joint, no ligamentous injury or osteochondral lesion	Soft tissue band extending from the anterior fibula (osteocartilaginous fossa) to medial gutter, adjacent area of chondromalacia	Arthroscopic debridement with a shaver	Protected weight-bearing in boot, return to full activity at 12 weeks
Rosenbaum et al., 2015 [7]	67-year-old female with no history of trauma	Chronic anterolateral ankle pain, refractory to steroid injection. Pain with forced dorsiflexion.	XR: Unremarkable; MRI: Synovitis in the anterolateral joint, osteochondral lesion at lateral talar dome (10 mm x 3 mm)	Plica extending from anterolateral synovitis to medial gutter, small osteochondral lesion (as seen on MRI)	Arthroscopic debridement (instrument not specified)	Weight-bearing not specified, return to full activity at 12 weeks

Highcock et al. described the first formal case report of ankle plica syndrome in a 16-year-old male soccer player with atraumatic persistent ankle pain and unremarkable MRI findings [5]. The authors were able to identify a suspected plica on MRI arthrogram, which was confirmed during arthroscopy. This plica, unlike prior reports, was medially based and was seen clearly draping over the anterior medial aspect of the talus, causing impingement during ankle flexion. Debridement resulted in complete symptom resolution. 

Somorjai et al. described an additional case shortly after in a 16-year-old male handball player [6]. In contrast to the previous case report by Highcocket al. [5], this patient reported a history of recurrent ankle sprains. The MRI findings suggested a possible flake fracture or loose body; however, arthroscopy identified an intra-articular plica originating from the osteochondral fossa at the anteromedial tibial plafond. The patient’s symptoms resolved after arthroscopic debridement [6]. 

Rosenbaum et al. [7] presented two patients with a different variation of plica than the previous two cases [5,6]. Their first patient was a 34-year-old female marathoner with a history of recurrent ankle sprains, and the second one was a 67-year-old female with no history of trauma. Both patients had chronic ankle pain and mechanical symptoms for which MRI was inconclusive. Both had failed conservative management with an intra-articular steroid injection. Arthroscopy revealed an intra-articular plica draping over the talus from medial to lateral. The authors believed that, due to the plica’s morphology, the plica was likely congenital in nature, and not due to injury or instability. Both patients experienced complete resolution of symptoms following surgical debridement [7]. 

The plica reported in our case was a discrete band of tissue that traversed the ankle joint from medial to lateral, potentially making it congenital in nature. Similar to the patients previously described with ankle plica syndrome, our patient had chronic pain with impingement symptoms refractory to conservative treatment. While half of the prior reported cases had a notable history of recurrent ankle sprains, our patient did not have a reported history of trauma. Furthermore, the plica in our case was seen as a distinctive soft tissue structure on MRI that correlated with arthroscopy findings. Many patients in the literature have additional findings of either extensive synovitis or adjacent chondromalacia. Our patient did not have any notable chondromalacia but did have extensive synovitis surrounding the insertion points of the plica as well as at the syndesmosis. Consistent with the outcomes in prior reports, the patient’s symptoms resolved shortly after arthroscopic debridement. 

## Conclusions

This case report highlights that ankle plicae can be a cause of painful ankle impingement in patients with chronic symptoms. Advanced imaging may be unclear, often showing synovitis with a thickened soft tissue density within the joint, which should alert clinicians to existence of a plica. For patients with ankle impingement symptoms refractory to conservative measures, ankle arthroscopy with debridement is a highly effective diagnostic and therapeutic tool. 
